# Upregulated GPRC5A disrupting the Hippo pathway promotes the proliferation and migration of pancreatic cancer cells via the cAMP-CREB axis

**DOI:** 10.1007/s12672-023-00626-1

**Published:** 2023-02-03

**Authors:** Weidan Fang, Xin Yu, Jun Deng, Bin Yu, Jianping Xiong, Mei Ma

**Affiliations:** 1 Department of Oncology, The First Affiliated Hospital of Nanchang University, Nanchang, 330006 Jiangxi Province China; 2 Jiangxi Key Laboratory for Individualized Cancer Therapy, Nanchang, China; 3 Department of General Surgery, The First Affiliated Hospital of Nanchang University, Nanchang, 330006 Jiangxi Province China

**Keywords:** Pancreatic cancer, GPRC5A, Hippo pathway, YAP1, cAMP-CREB

## Abstract

**Background:**

Pancreatic cancer has a high mortality rate worldwide, and is predicted to be third leading cause of death in the near future. However, the regulatory mechanisms that inhibit the progression of pancreatic cancer remain elusive. Currently, exploring the function and mechanisms of GPCRs (G-protein coupled receptors) is an important way to discover promising therapeutic targets for cancer.

**Methods:**

GPRC5A expression was measured using real-time quantitative PCR, immunohistochemistry and western blot assays. Cell proliferation and migration were assessed using CCK-8, clone formation, wound-healing and transwell assays. A cytosolic/nuclear distribution experiment was used to detect the protein location transfer. A xenograft model of pancreatic cancer was established to explore the role of GPRC5A in vivo.

**Results:**

GPRC5A expression was increased in pancreatic cancer, and disruption of GPRC5A expression inhibited tumor growth in vivo. Mechanistically, GPRC5A positively regulated the transcription of YAP1 through cAMP-CREB signaling. Moreover, we show that the proliferation and migration induced by GPRC5A in pancreatic cancer could be rescued by inhibiting YAP1 expression.

**Conclusions:**

GPRC5A interacts with the Hippo pathway to promote the progression of pancreatic cancer. These findings reveal an important crosstalk model and provide potential targets for pancreatic cancer therapy.

**Supplementary Information:**

The online version contains supplementary material available at 10.1007/s12672-023-00626-1.

## Background

The incidence of pancreatic cancer is increasing around the world [1]. Due to its high mortality rate, pancreatic cancer is currently the seventh leading cause of cancer deaths worldwide and may move up to the third in the near future [2]. Pancreatic ductal adenocarcinoma (PDAC) presently makes up the main diagnosed cases. As it is a fatal malignancy [3], it is urgent for more basic research to find new targets and bring new hope for pancreatic cancer treatment.

G-protein coupled receptors (GPCRs) are one of the largest membrane families that mediate extracellular signal transduction into cells and play a key role in many cell functions. GPCRs are one of the most promising therapeutic targets for cancer and targeting GPCR-disturbed cell signal transduction has become an important strategy for cancer drug development [4, 5]. Recent studies have shown that GPRC5A (G-Protein coupled Receptor Class C, Group 5, Member A) functions in the progression of a variety of cancer, including pancreatic cancer [6]. The expression and function of GPRC5A vary in cancers. For example, GPRC5A expression is reduced in lung cancer and inhibits lung tumorigenesis [7, 8], while in most cancers, GPRC5A functions as an oncogene. In pancreatic cancer, GPRC5A can indirectly interact with the RNA-binding protein HuR, which influences its translation [9]. GPRC5A may change the phosphorylation levels of STAT3 [10] and GSK-3β [11], through there is no evidence demonstrating that inhibiting the phosphorylation of these proteins blocks GPRC5A function. Therefore, it is still important to determine the key downstream molecules of GPRC5A and its mechanism of action in pancreatic cancer.

It is widely recognized that Hippo signaling is involved in the tumor suppressor pathway [12]. The transcriptional coactivator YAP1 is the core protein of the Hippo pathway, and its nuclear/cytoplasmic distribution is regulated by a serine/threonine kinase cascade that is launched through Hippo signaling activity [13]. Dysregulation of the Hippo pathway in pancreatic cancer is well known [14]. Multiple genes regulated by YAP1 are related to the unfavorable survival of pancreatic cancer patients, such as FOSL1 [15], CCND1 [16], and NOTCH2 [17]. GPCRs are an important type of upstream regulator that activate or inhibit the Hippo pathway [18]. In hypoxic situations, it has been reported that HIFs can reduce the phosphorylation level of YAP1 by directly promoting GPRC5A transcription [19]. However, the interaction between GPRC5A and YAP1 in pancreatic cancer is unclear.

In this study, we first address the roles of GPRC5A in nude mice and show that its overexpression in pancreatic cancer is predictive of poor prognosis. We demonstrate that GPRC5A regulates YAP1 expression at the transcription level but not at the activity level, which is governed by the phosphorylation kinase MST1/2 and LATS1/2. Importantly, YAP1 knockdown reversed the adverse effects of GPRC5A on pancreatic cancer cells. Moreover, we observed GPRC5A-influenced expression by cAMP-CREB signaling. We propose that the GPRC5A-cAMP-CREB axis is a crucial pathway in the promotion of pancreatic cancer progression.

## Materials and methods

### Cell lines and cell culture

The pancreatic cancer cell lines AsPC-1, BxPC-3, PANC1 and MIA PaCa-2 and human pancreatic ductal epithelial cell line hTERT-HPNE were obtained from the Beijing Beina Chuanglian Institute of Biotechnology (Beijing, China), cultured in DMEM (HyClone, Logan, UT, USA) containing 10% fetal bovine serum (FBS, HyClone, USA) and incubated at 37 °C with 5% CO_2_.

### Plasmid, lentiviral infection, and transfection

The pcDNA3.1-GPRC5A plasmid was kindly provided by Prof. Jiong Deng (Shanghai Jiao Tong University, Shanghai, China). The GPRC5A plasmid (CMV-GPRC5A-EF1A-EGFP), GPRC5A knockdown lentivirus vector hU6-sh-GPRC5A-Ubi-EGFP (LV-sh-GPRC5A), and hU6-sh-Con-Ubi-EGFP empty lentiviral vector (LV-sh-NC) were synthesized by Genechem (Shanghai, China) [20]. The YAP1 plasmid (CMV-YAP1-T2A-EGFP), YAP1-targeting siRNA oligonucleotides were designed and synthesized by GenePharma (Shanghai, China). Cells were seeded on the plate one day before transfection and transfected using TurboFect transfection reagent (R0532; Thermo Scientific Scientific, Waltham, MA, United States). PANC1, MIA PaCa-2 and BxPC-3 cells were infected with lentivirus according to the manufacturer’s instructions. The sequences of the shRNA and siRNA used in our study were as follows: LV-sh-GPRC5A: 5’-CCTGACCATGAATAGGACCAA-3’, LV-sh-Con: TTCTCCGAACGTGTCACGT; si-YAP1: 5’-GGUGAUACUAUCAACCAAATT-3’, si-Con: UUCUCCGAACGUGUCAUGUTT.

### Western blotting analysis

Total protein was extracted using RIPA buffer containing a protease inhibitor cocktail and phosphatase inhibitor cocktail (CWBIO, Jiangsu, China), separated by 10% SDS-PAGE, and then transferred to polyvinylidene difluoride membranes. The protein marker used in Western blotting was obtained from ThermoFisher Scientific (26616; ThermoFisher Scientific, Waltham, MA, United States). The membranes were blocked in 5% skim milk for 1 h at room temperature and then incubated at 4 °C with primary antibodies against GPRC5A (1:500; SC-373824; Santa Cruz), MST1 (1:500; ab51134; Abcam), MST2 (1:1000; #3952; Cell Signaling Technology), p-MST1/2 (1:500; 28953-1-AP; Proteintech), LATS1 (1:500; SC-398560; Santa Cruz), LATS2 (1:1,000; ab110780; Abcam), p-LATS1/2 (1:1,000; AF8163; Affinity), YAP1 (1:1,000; ab52771; Abcam), CYR61 (1:2,000; #14479; Cell Signaling Technology), Cyclin D1 (1:2,000; #2978; Cell Signaling Technology), c-Myc (1:2,000; #5605; Cell Signaling Technology), CTGF (1:2,000; #86641; Cell Signaling Technology), CREB (1:1,000; 13584-1-AP Proteintech), p-CREB (1:1,000; AP0019; ABclonal), GAPDH (1:10,000; 60004-1-Ig; Proteintech), or Histone-H3 (1:2,000; 17168-1-AP; Proteintech) overnight. The membranes were then washed three times with Tris-buffered saline with 0.1% Tween-20 (TBST buffer) and incubated for 1 h at room temperature with horseradish peroxidase (HRP)-conjugated secondary antibodies (ZSGB, China). After three final TBST washes, the resulting signals were detected via a chemiluminescence solvent (Thermo Fisher Scientific, Waltham, MA, USA).

### Immunohistochemistry

Paraffin-embedded human pancreatic cancer tissues and adjacent normal tissues were collected from patients treated at the First Affiliated Hospital of Nanchang University. This study was approved by the Ethics Committee of the First Affiliated Hospital of Nanchang University. All paraffin-embedded tissues were deparaffinized with xylene, rehydrated with graded concentrations of alcohol, and subjected to antigen retrieval with citrate buffer. Endogenous peroxidase activity was blocked with 3% H_2_O_2_. Paraffin sections were incubated overnight with primary anti-GPRC5A or anti-YAP1 antibodies. Next, the sections were incubated with the appropriate secondary antibody, and DAB solution was added. All staining scores were assessed by two pathologists in a blinded manner based on staining intensity and positive staining ratio, and the grading standards were carried out as previously described [21].

### RNA isolation and real-time quantitative PCR

Total RNA was extracted from cells or xenograft tumor tissues using TRIzol (Invitrogen, Carlsbad, CA, USA) according to the manufacturer’s protocol. cDNA was obtained by reverse transcription using PrimeScript™ RT reagent Kit with gDNA Erase (RR047A, Takara, Dalian, China). Real-time quantitative PCR (RT-PCR) was performed on an ABI Step One Plus system (Applied Biosystems, Foster City, CA, USA) using TB Green™ Premix Ex Taq II (RR820A, Takara, Dalian, China). Relative gene expression levels were normalized to GAPDH and calculated using the 2^−ΔΔCT^ method. The primer sequences used are listed in Table 1.

**Table 1:** Primers used for the RT-PCR analyses of mRNA expression

Gene	Forward primers	Reverse primers
GPRC5A	TCTATGCCCCCTATTCCACA	TGCATTTGTCCCACTCTTCA
YAP1	TCGTTTTGCCATGAACCAGA	GGCTGCTTCACTGGAGCACT
CYR61	CGAGGTGGAGTTGACGAGAA	GCACTCAGGGTTGTCATTGGT
CyclinD1	CAGAGGCGGAGGAGAACAAA	ATGGAGGGCGGATTGGAA
CTGF	GCGAAGCTGACCTGGAAGAGAAC	GGCCGTCGGTACATACTCCAC
c-MYC	AGCCTCCCGCGACGAT	GAGTCGTAGTCGAGGTCATAGTTCCT
cAMP	ATGCTAACCTCTACCGCCTCCTG	ATGCTAACCTCTACCGCCTCCTG
GAPDH	ATCATCCCTGCCTCTACTGG	TGGGTGTCGCTGTTGAAGTC

### RNA sequencing and analysis and bioinformatics analysis

Total RNA was extracted from PANC1 cells in the LV-sh-NC group or LV-sh-GPRC5A group using TRIzol Reagent. Partly isolated RNA samples were submitted to Beijing Genomics Institute for transcriptome sequencing following standard protocols and analysis on BGISEQ-500 [22]. Standard bioinformatics analysis was performed by the Beijing Genomics Institute. For gene expression analysis, the significance of the differentially expressed genes (DEGs) was defined by the bioinformatics service of BGI according to the combination of the absolute value of |log2(FC)|> 1 and adjusted p value < 0.05. GEPIA (http://gepia.cancer-pku.cn/) was used to determine and assess the expression and prognostic values of GPRC5A in pancreatic cancer. UALCAN (http://ualcan.path.uab.edu) was used to analyze the associations of GPRC5A with cancer stage and nodal metastasis status in pancreatic cancer. A p value < 0.05 was considered to be statistically significant.

### Cell counting kit-8 (CCK-8) assay

Cells were plated in 96-well plates with 1 × 10^3^ cells per well. Next, 100 μl FBS-free medium containing 10% CCK8 reagent was added to each well and incubated for 2 h at 37 °C. Finally, the absorbance of the cells was measured using a Varioskan LUX™ multimode microplate reader (Thermo Scientific) at 450 nm for 5 days. These experiments were conducted more than three times.

### Colony formation assay

Cells were plated in 6-well plates with 2 × 10^3^ cells per well. After 14 days, the colonies were fixed with methanol and stained with 1% crystal violet. The number of colonies was evaluated using ImageJ software.

### Wound-healing assay

Cells were plated into 6-well plates with 5 × 10^5^ cells per well until completely confluent. Straight wounds were scratched using a sterile 10 μl pipette tip and washed with phosphate-buffered saline three times. Next, the cells were incubated in serum-free medium for 24 h. Images were taken at 0 h and 24 h to calculate the wound closure percentage. Wound closure ranges in three randomly selected microscopic fields were counted for each group.

### Transwell assays

Cells were plated into the upper compartment of the transwell chamber with 4 × 10^4^ cells per well in 200 μl serum-free medium. The bottom chamber contained 600 μl DMEM with 20% FBS. After 48 h, cells that migrated through the chamber membranes were fixed with methanol and stained with 1% crystal violet.

### Animal models

All animal experiments were performed using protocols approved by the Animal Center of The First Affiliated Hospital of Nanchang University. Six-week-old female nude mice were purchased from Hunan SJA Laboratory Animal Co., Ltd. (Hunan, China), and 1 × 10^7^ LV-sh-NC or LV-sh-GPRC5A BxPC-3 cells were subcutaneously inoculated into the left axilla of each mouse. Tumor growth was measured every week with electronic digital calipers (Thermo Scientific). Tumor volume was calculated with the formula: tumor volume (mm^3^) = [length × (width)^2^]/2. After 5 weeks, the xenograft tumors were harvested and weighed.

### Statistical analysis

All the data are presented as the mean ± standard deviation. The Student’s t-test, one-way ANOVA, two-way ANOVA, and log-rank (Mantel-Cox) test were adopted for the statistical analyses. P < 0.05 was considered statistically significant. * indicates P < 0.05, ** indicates P < 0.01, and *** indicates P < 0.001.

## Results

### Upregulation of GPRC5A predicts poor prognosis in pancreatic cancer

**Fig. 1:**
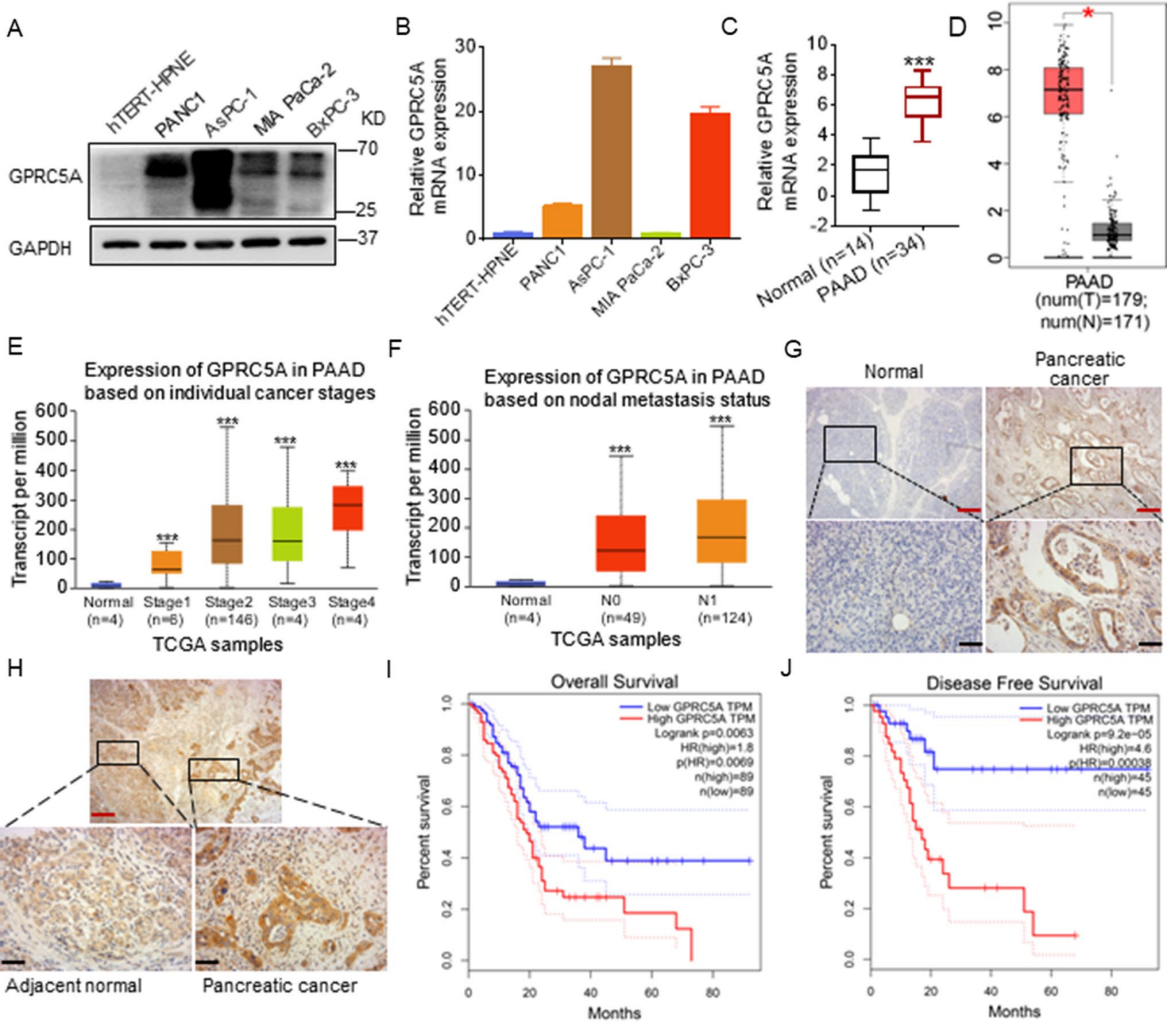
GPRC5A is upregulated in pancreatic cancer and predicts poor prognosis in pancreatic cancer patients. **A, B** The protein (**A**) and mRNA (**B**) expression level of GPRC5A in pancreatic cancer cell lines and normal hTERT-HPNE cells by Western blotting and RT-PCR. **C, D** Analysis of GPRC5A mRNA relative expression in the TCGA (**C**) and GEPIA (**D**) databases. Student’s t test, *p < 0.05, ***p < 0.001. **E, F** Association of GPRC5A expression with cancer stage (**E**) and nodal metastasis status (**F**) in pancreatic cancer patients. One-way ANOVA, post hoc, Bonferroni, ***p < 0.001. **G, H** Representative images of IHC staining of GPRC5A in human pancreatic cancer tissues and normal tissue (**G**) and adjacent normal tissue samples (**H**). Red lines represent 200 μm, and blank lines represent 50 μm. **I, J** The overall survival (**I**) and disease-free survival (**J**) of pancreatic cancer patients treated with GPRC5A were obtained using the GEPIA database. Log-rank (Mantel-Cox) test

To further demonstrate the role of GPRC5A in pancreatic cancer, we detected its expression in pancreatic cancer cells and tissues. We first examined GPRC5A expression in pancreatic cancer cell lines and normal cells at the protein and mRNA levels, and observed that GPRC5A expression was higher in pancreatic cancer cells than in normal cells (Fig. 1A, B). GPRC5A was significantly upregulated in pancreatic cancer compared to normal tissues analyzed through the TCGA and GEPIA databases (Fig. 1C, D). Based on cancer stages, compared with the normal group, we found that patients in higher stages had more GPRC5A expression. In addition, an increasing trend of GPRC5A expression was observed from stage I to stage IV (Fig. 1E). In terms of nodal metastasis status, both N0 and N1 had significantly greater expression of GPRC5A than the normal group (Fig. 1F). In addition, we observed increased GPRC5A expression in the tumor tissues in contrast to normal tissues in pancreatic cancer patients by IHC (Fig. 1G). Similarly, the GPRC5A protein level was higher in pancreatic cancer tissues than in adjacent normal tissues (Fig. 1H). Last, we found that upregulated GPRC5A predicted poor overall survival (OS) and disease-free survival (DFS) using the GEPIA database (Fig. 1I, J). Thus, these results suggested that GPRC5A is overexpressed in pancreatic cancer tissues and cells and predicts a poor prognosis for pancreatic cancer patients.

### Knockdown of GPRC5A inhibits cell proliferation and migration of pancreatic cancer in vitro

**Fig. 2:**
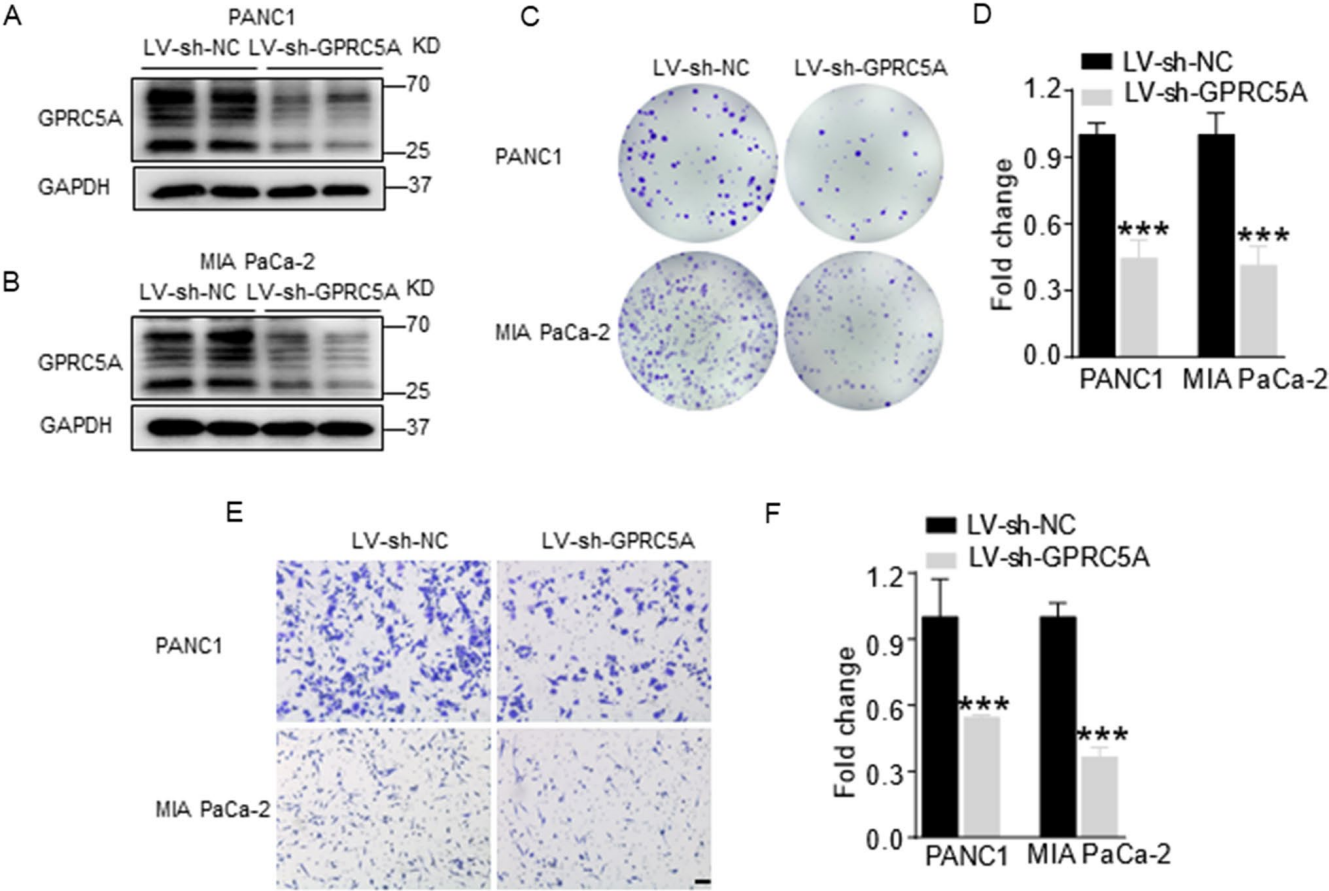
Knockdown of GPRC5A inhibits cell proliferation and migration of pancreatic cancer in vitro. **A, B** PANC1 and MIA PaCa-2 cells were transfected with GPRC5A shRNA, and the efficiency was detected by Western blotting. **C, D** Colony formation assay was used to evaluate cell proliferation. Student’s t test, ***p < 0.001. **E, F** Transwell assay was used to evaluate migration ability. Scale bar = 50 μm. Student’s t test, ***p < 0.001

To explore the efficiency of GPRC5A shRNA, the whole transcriptome profiles of PANC1 cells with LV-sh-NC or LV-sh-GPRC5A were analyzed by RNA-seq and confirmed in PANC1 and MIA PaCa-2 cells by Western blotting (Fig. 2A, B). Then, we performed functional experiments to assess the role of GPRC5A in pancreatic cancer cells. Colony formation assays revealed that GPRC5A knockdown markedly decreased cell growth in PANC1 and MIA PaCa-2 cells (Fig. 2C, D). Interestingly, GPRC5A knockdown also markedly decreased the migration ability of pancreatic cancer cells (Fig. 2E, F). Taken together, our data indicate that GPRC5A knockdown suppresses pancreatic cancer cell proliferation and migration in vitro.

### GPRC5A promotes the growth of pancreatic cancer in vivo

**Fig. 3:**
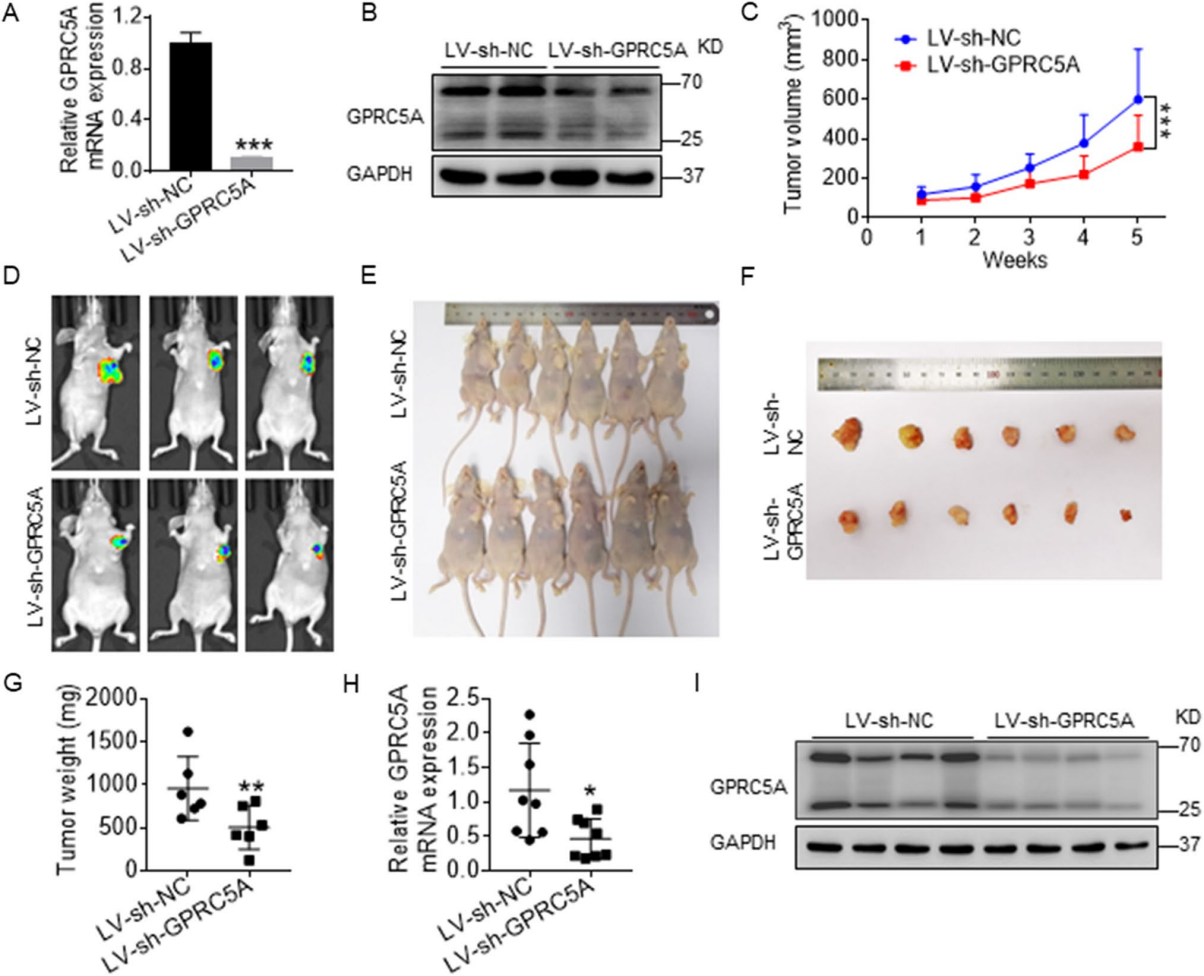
GPRC5A promotes the growth of pancreatic cancer in vivo. **A, B** The knockdown efficiency was detected by RT-PCR and Western blotting in BxPC-3 cells infected with LV-sh-NC or LV-sh-GPRC5A. Student’s t test, ***p < 0.001. **C** Weekly volume change in nude mice after tumors implantation. Two-way ANOVA, post hoc, Bonferroni, ***p < 0.001. **D–F** Tumor volume images are shown in the LV-sh-GPRC5A group and LV-sh-NC group in nude mice at the fifth week. n = 6. **G** Quantification of the average weights of tumor tissues dissected from the nude mice. Student’s t test, **p < 0.01. **H, I** The mRNA and protein levels of GPRC5A were detected by RT-PCR and Western blotting. Student’s t test, *p < 0.05

To investigate the effect of GPRC5A on tumor growth, we next established a subcutaneous xenograft tumor model. Pancreatic cancer cells Infected with LV-sh-NC or LV-sh-GPRC5A were inoculated into nude mice, and tumor size was monitored for 5 weeks. The knockdown efficiency of GPRC5A was analyzed using RT-PCR and Western blotting (Fig. 3A, B). The volume of the tumors was significantly reduced in the LV-sh-GPRC5A group compared to the LV-sh-NC group from the first week to the fifth week (Fig. 3C). After 5 weeks of implantation, the tumor parts were directly inspected or dissected from the nude mice to measure the volume and weight (Fig. 3D–F). The tumor weight was significantly decreased in the LV-sh-GPRC5A group as opposed to the LV-sh-NC group in the fifth week (Fig. 3G). To confirm the expression of GPRC5A, xenograft tumors were identified by performing RT-PCR and Western blotting. As expected, the mRNA and protein levels of GPRC5A were significantly decreased in the LV-sh-GPRC5A group (Fig. 3H, I). Collectively, our data demonstrate that GPRC5A knockdown inhibits the growth of implanted tumors in vivo.

### GPRC5A dysregulates the Hippo pathway by regulating the YAP1 transcript

**Fig. 4:**
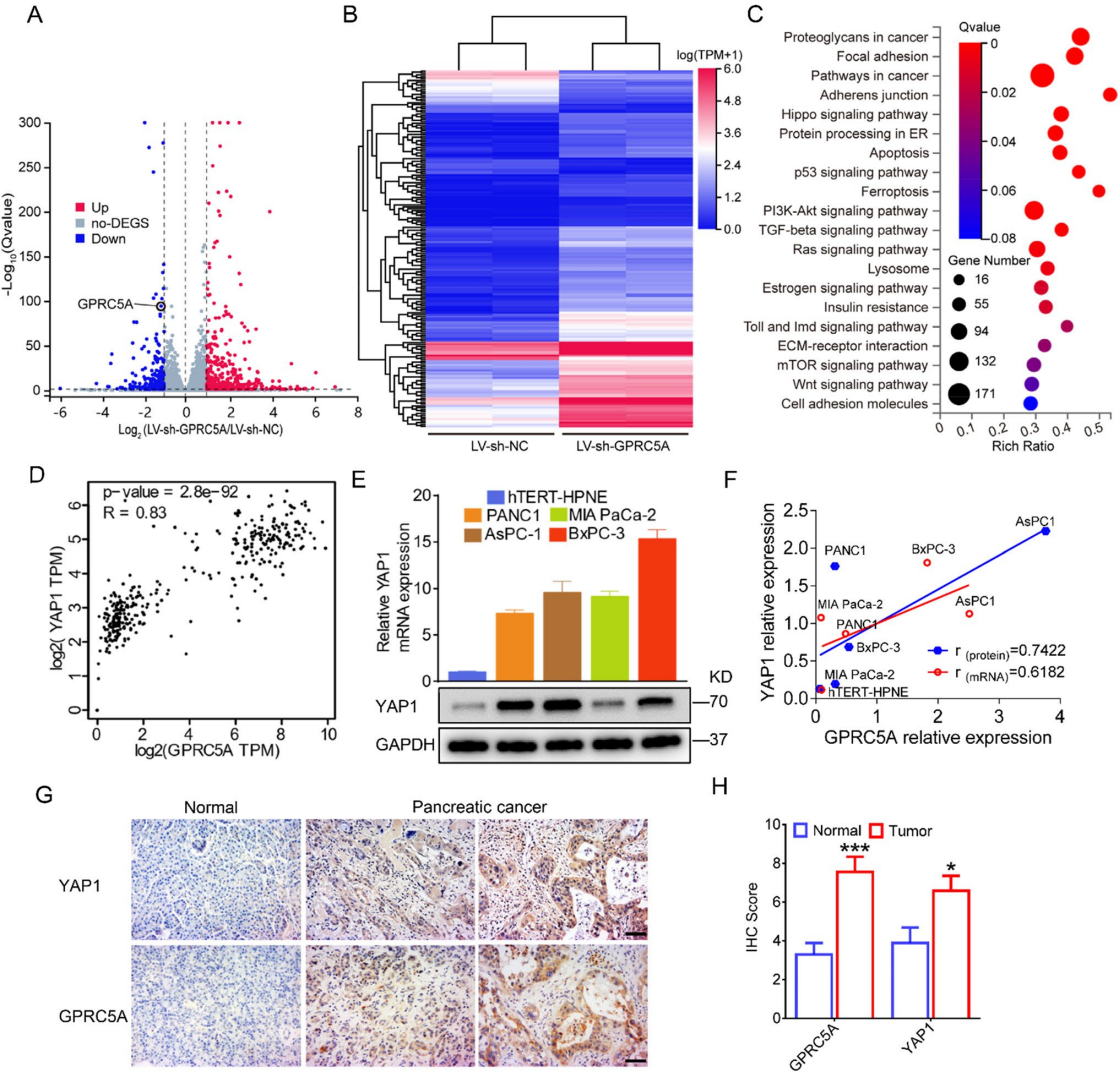
GPRC5A is positively correlated with YAP1 in pancreatic cancer. **A** The volcano plot presents the GPRC5A-related differentially expressed genes (DEGs) by RNA sequencing analysis. Red and green dots represent up and downregulated genes, respectively. **B** Heat map and hierarchical clustering based on the most differentially expressed genes screened out with the criteria of |log2(FC)|> 2 and adjusted p value < 0.05. **C** KEGG pathway enrichment analysis was used to identify the top 20 cancer-related pathways with significant enrichment. **D** The correlation between GPRC5A and YAP1 in pancreatic cancer was evaluated using the GEPIA database. **E** The protein and mRNA expression levels of YAP1 in pancreatic cancer cell lines and normal hTERT-HPNE cells by Western blotting and RT-PCR. **F** The correlation between GPRC5A and YAP1 was detected in the mRNA and protein levels in pancreatic cancer cell lines and normal hTERT-HPNE cells. **G** Representative images of GPRC5A and YAP1 in human pancreatic cancer tissues and adjacent normal tissue samples as shown by IHC staining. Scale bar = 50 μm. **H** The IHC scores of GPRC5A and YAP1 in human pancreatic cancer tissues. Student’s t test, *p < 0.05, ***p < 0.001

To explore the potential molecular mechanism that was responsible for the effects of GPRC5A on pancreatic cancer progression, the whole transcriptome profiles of PANC1 cells with LV-sh-NC group or LV-sh-GPRC5A group were analyzed by RNA-seq. We screened out DEGs in LV-sh-GPRC5A group compared to the LV-sh-NC group with the criteria of |log2(FC)|> 1 and adjusted p value < 0.05, which included 413 upregulated genes and 247 downregulated genes (Fig. 4A). Among them, we further selected 192 DEGs with the criteria of |log2(FC)|> 2 displayed on the heat map (Fig. 4B). KEGG enrichment analyses showed that the DEGs were enriched in cancer-related pathways, including Hippo signaling pathway, p53 signaling pathway, PI3K-Akt signaling pathway TGF-beta signaling pathway (Fig. 4C). Disruption of Hippo signaling is common in pancreatic cancer [14], and has an important influence on pancreatic cancer tumorigenesis and progression [23, 24]. Previous research has shown that GPRC5A promotes cancer cell adaptation to hypoxia by activating YAP1, the core protein of the Hippo pathway [19]. To investigate whether GPRC5A promoted pancreatic cancer progression via the YAP1 pathway, we evaluated the relationship between GPRC5A and YAP1 in pancreatic cancer tissues and cells. We first found a significantly positive relationship between GPRC5A and YAP1 in pancreatic cancer using the GEPIA database (Fig. 4D). Next, we examined YAP1 expression in pancreatic cancer cell lines and normal cells at both the mRNA and protein levels and found that the expression of YAP1 was positively related to GPRC5A expression (Fig. 4E, F). Correspondingly, GPRC5A and YAP1 had similar expression patterns in tumor tissues (Fig. 4G, H).

**Fig. 5:**
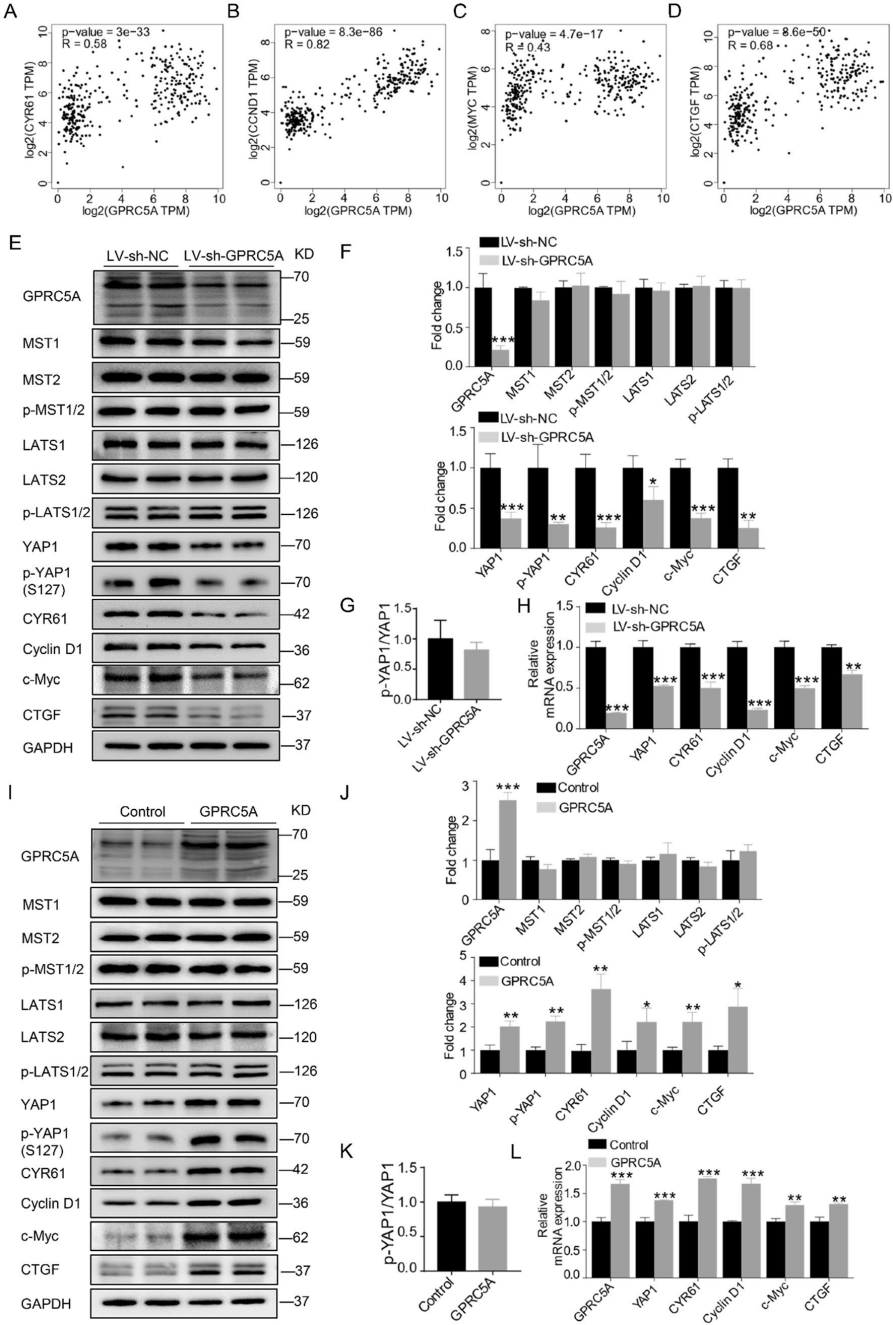
GPRC5A modulates the Hippo pathway by mediating YAP1 transcription. **A–D** The correlation between GPRC5A and CYR61, cyclin D1, c-Myc and CTGF in pancreatic cancer was evaluated using the GEPIA database. **E**, **F** Western blotting analysis of MST1/2, p-MST1/2, LATS1/2, p-LATS1/2, YAP1, p-YAP1, CYR61, cyclin D1, c-Myc and CTGF expression after GPRC5A downregulation. Student’s t test, *p < 0.05, **p < 0.01, ***p < 0.001. **G** The ratio between YAP1 and p-YAP1 after downregulation of GPRC5A. **H** RT-PCR analysis of YAP1, CYR61, cyclin D1, c-Myc and CTGF after downregulation of GPRC5A. Student’s t test, *p < 0.05, **p < 0.01, ***p < 0.001. **I-J** Western blotting analysis of MST1/2, p-MST1/2, LATS1/2, p-LATS1/2, YAP1, p-YAP1, CYR61, cyclin D1, c-Myc and CTGF expression after GPRC5A upregulation. Student’s t test, *p < 0.05, **p < 0.01, ***p < 0.001. **K** The ratio between YAP1 and p-YAP1 after upregulation of GPRC5A. **L** RT-PCR analysis of YAP1, CYR61, cyclin D1, c-Myc and CTGF after upregulation of GPRC5A. Student’s t test, *p < 0.05, **p < 0.01, ***p < 0.001

To explore the impact of GPRC5A on YAP1, we evaluated the relationship between GPRC5A and YAP1 downstream target genes in pancreatic cancer patients. The results showed that GPRC5A was closely correlated with CYR61, cyclin D1, c-Myc, and CTGF in pancreatic cancer (Fig. 5A–D). We then examined the protein expression of the Hippo pathway after bidirectional control of GPRC5A. The results showed that knockdown or overexpression of GPRC5A prominently decreased or increased, respectively, the expression of YAP1 and its target genes, CYR61, cyclin D1, c-Myc and CTGF (Fig. 5E, F, I, J). Regulation of GPRC5A expression did not impact on MST1/2, LATS1/2 and its phosphorylated states, which is the upstream kinase that regulates the YAP1 activity [25] (Fig. 5E, F, I, J). In addition, YAP1 and p-YAP1 changed synchronously without a significant change in the ratio between YAP1 and p-YAP1 based on the regulation of GPRC5A expression (Fig. 5G, K). Moreover, we examined the mRNA expression of YAP1 and its downstream genes based on modulation of GPRC5A expression. The results showed that GPRC5A positively regulated YAP1, CYR61, cyclin D1, c-Myc and CTGF expression at the transcriptional level, as evidenced by overexpression and knockdown of GPRC5A in pancreatic cancer cells (Fig. 5H, L). Taken together, our results show that GPRC5A dysregulates Hippo signaling by regulating YAP1 transcription.

### YAP1 interference inhibits the effects of GPRC5A on the proliferation and migration of pancreatic cancer cells

**Fig. 6:**
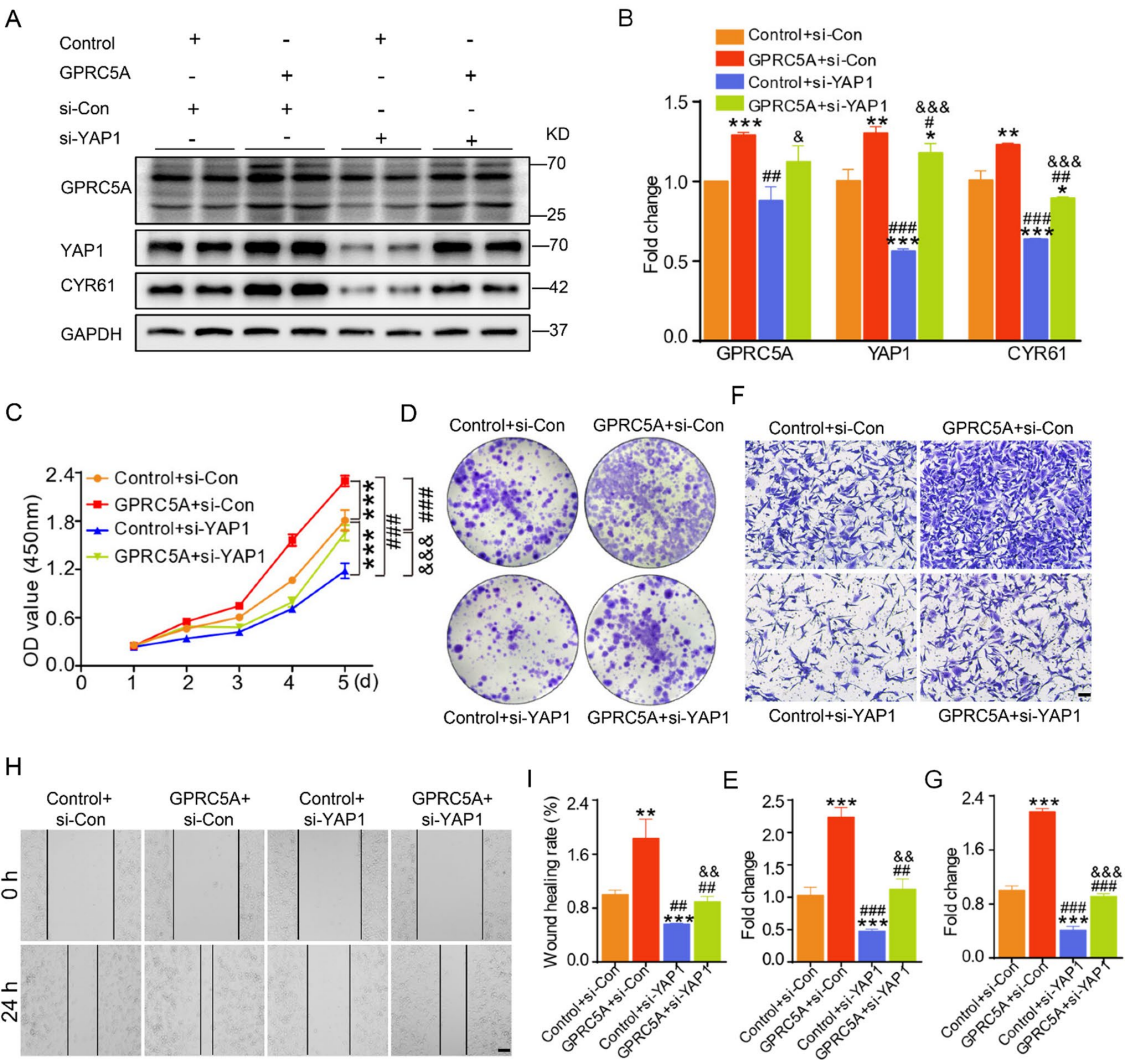
YAP1 interference inhibits the effects of GPRC5A on the proliferation and migration of pancreatic cancer cells. **A, B** PANC1 cells were transfected with GPRC5A or control plasmids combined with YAP1 siRNA or si-Con, and Western blotting was performed to detect the expression of GPRC5A, YAP1 and its target gene CYR61. One-way ANOVA, post hoc, Bonferroni, *p < 0.05, **p < 0.01, ***p < 0.001, (vs. Control + si-Con); #p < 0.05, ##p < 0.01, ###p < 0.001, (vs. GPRC5A + si-Con); &p < 0.05, &&&p < 0.001, (vs. Control + si-YAP1). **C–E** Cell proliferation was evaluated using CCK-8 (**C**) and colony formation assays (**D**, **E**) performed on GPRC5A-overexpressing cells with YAP1 knockdown. Two-way ANOVA and one-way ANOVA, post hoc, Bonferroni, ***p < 0.001, (vs. Control + si-Con); ##p < 0.01, ###p < 0.001, (vs. GPRC5A + si-Con); &&p < 0.01, &&&p < 0.001, (vs. Control + si-YAP1). **F–I** Cell migration ability was evaluated via transwell (**F**, **G**) and wound-healing assays (**H**, **I**) after GPRC5A overexpression with YAP1 knockdown. Scale bar = 50 μm. One-way ANOVA, post hoc, Bonferroni, **p < 0.01, ***p < 0.001, (vs. Control + si-Con); ##p < 0.01, ###p < 0.001, (vs. GPRC5A + si-Con); &&p < 0.01, &&&p < 0.001, (vs. Control + si-YAP1)

To further determine the role of YAP1 in GPRC5A-mediated function in pancreatic cancer, we upregulated YAP1 using YAP1 plasmid with or without downregulation of GPRC5A in pancreatic cancer cells. Upregulating YAP1 only increased its expression without influencing GPRC5A expression in PANC1 cells (Supplementary Figure S1A, B). In addition, YAP1 overexpression partially counteracted the effects of GPRC5A knockdown (Supplementary Figure S1A, B). Interestingly, we downregulated YAP1 using siRNA with or without GPRC5A overexpression in pancreatic cancer cells. Disrupting YAP1 only reduced its expression without influencing GPRC5A expression in PANC1 cells (Fig. 6A, B). In addition, YAP1 deficiency partially counteracted the effects of GPRC5A upregulation of YAP1 and its target gene CYR61 (Fig. 6A, B). Furthermore, the influence of GPRC5A on promoting the proliferation and migration of pancreatic cancer cells was partially reversed by YAP1 deficiency in PANC1 cells, which was verified by CCK-8, colony formation, transwell, and wound healing assays (Fig. 6C–I). In summary, our findings demonstrated that inhibiting YAP1 was beneficial in disrupting the adverse effects of GPRC5A on pancreatic cancer cells.

### GPRC5A mediates the Hippo-YAP1 pathway through cAMP-CREB signaling

**Fig. 7:**
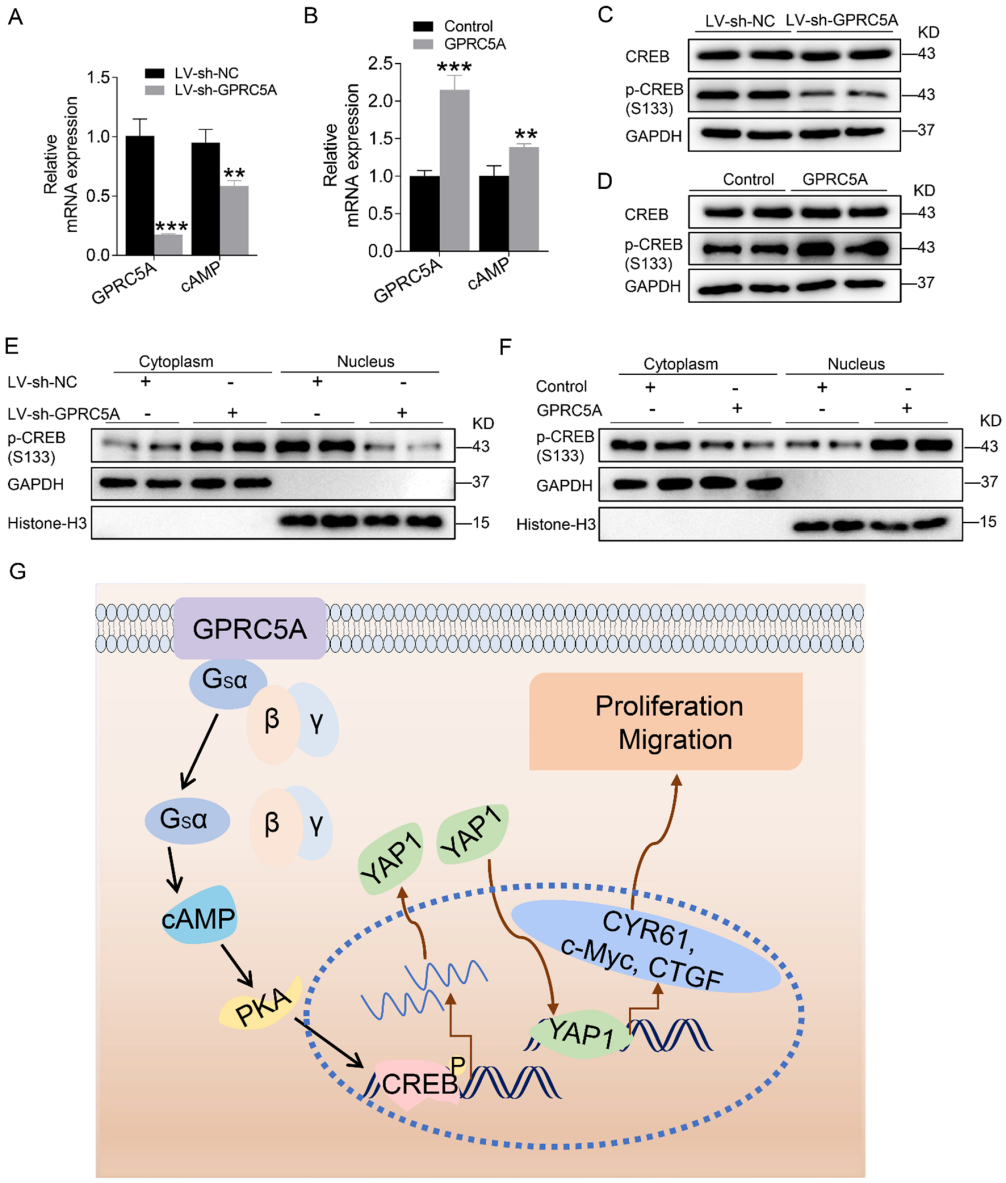
GPRC5A mediates the Hippo-YAP1 pathway through the cAMP-CREB axis. **A, B** RT-PCR analysis of cAMP expression after GPRC5A was downregulated or upregulated. Student’s t test, **p < 0.01, ***p < 0.001. **C, D** Western blotting was performed to detect p-CREB and CREB expression following GPRC5A knockdown or overexpression. **E, F** Western blotting showing the distribution of p-CREB in the cytoplasmic and nuclear extracts follwing modification of GPRC5A expression in pancreatic cancer cells. **G** The proposed working model of GPRC5A promoting pancreatic cancer cell proliferation and migration by disrupting the Hippo pathway through cAMP-CREB signaling

It has been previously reported that GPCRs promote the generation of cAMP (cyclic adenosine monophosphate), which is vital for the progression of physiology and pathology [26, 27]. Thus, we investigated cAMP expression after regulating GPRC5A in pancreatic cancer cells. The results showed GPRC5A knockdown decreased the mRNA expression of cAMP (Fig. 7A). Moreover, GPRC5A overexpression increased cAMP expression (Fig. 7B). cAMP-PKA-CREB signaling plays an important role in cancer cell growth, migration, and metabolism [28]. CREB is activated by Ser133 phosphorylation and then binds to YAP1 promoter areas to upregulate its transcription [29, 30] Therefore, we investigated whether GPRC5A controlled YAP1 expression by cAMP/CREB pathways. To test this hypothesis, we first measured the effect of GPRC5A on CREB activity, and our results indicated that silencing or overexpressing GPRC5A drastically increased or decreased the phosphorylation of CREB without significantly affecting total CREB expression (Fig. 7C, D). Intriguingly, we observed that nuclear localization of phosphorylated CREB was upregulated and cytoplasmic phosphorylated CREB expression was downregulated upon GPRC5A overexpression (Fig. 7F). Conversely, GPRC5A knockdown decreased nuclear phosphorylated CREB levels and promoted cytoplasmic phosphorylated CREB expression (Fig. 7E). Taken together, these data suggested that GPRC5A disrupted the Hippo-YAP1 pathway through cAMP-CREB signaling (Fig. 7G).

## Discussion

Aberrant expression of GPRC5A is usually positive or negative in controlling the process of tumorigenesis [6], an effect that depends on its expression level and downstream regulatory molecules. Approximately half of the studies on GPRC5A have focused on the lung, and GPRC5A acts as a tumor suppressor in lung cancer with low expression [31, 32]. In most reported cancers, GPRC5A promotes cancer progression regardless of its up- or downregulation in cells [6]. In our experiments, GPRC5A was markably increased in pancreatic cancer compared to normal or adjacent tissue, and we first confirmed that in pancreatic cancer, GPRC5A interference inhibited tumor growth in nude mice.

Although two papers have explored GPRC5A function in pancreatic cancer [10, 11], they did not provide evidence from in vivo experiments or show that blocking downstream molecules disrupted the effect of GPRC5A. Our experiments showed that GPRC5A could bidirectionally control the expression of YAP1 and its target genes at the transcriptional level in pancreatic cancer cells. However, this is very different from studies in hypoxic conditions, which showed that GPRC5A influences the phosphorylation of LATS1/2 kinases to regulate YAP1 activity [19]. YAP1 activity, particularly its phosphorylation level, is commonly regulated by the LATS1/2 kinase of the Hippo pathway and is critical for tumor initiation, progression, and metastasis [33, 34]. Our results suggest that MST1/2 and LATS1/2 protein did not change markedly based on modulation of GPRC5A expression, which indicated that the regulation of YAP1 by GPRC5A is independent of LATS1/2 kinase in pancreatic cancer cells. Furthermore, our data showed that silencing YAP1 by siRNA disrupted the malignant biological behavior of GPRC5A in pancreatic cancer cells. These findings reveal an important crosstalk model between GPRC5A and the Hippo pathway in pancreatic cancer progression.

Activation of GPCRs always results in increased cAMP [35]. CREB activity is a marker of cAMP accumulation and a prominent direct target of PKA. It is well known that cAMP-PKA-CREB signaling participates in tumorigenesis [28]. Here, we demonstrated that GPRC5A upregulated cAMP and promoted CREB phosphorylation. One previous study showed that the cAMP-PKA axis also phosphorylates YAP1, through the response time lasted only 1 h ~ 4 h [36]. Our experiments demonstrated that disrupting YAP1 expression almost completely inhibited the effect of GPRC5A on pancreatic cancer cells. GPCRs play important roles in pathophysiology and pharmacological tractability; thus, GPCRs have been deemed to be one of the most valuable therapeutic targets for cancer treatment [37]. Moreover, the success rates of GPCR agents in phases I, II, and III were moderately higher than FDAs-approved average for all examined agents. However, compounds that disrupt GPCR-mediated pathways are limited in clinical application [4]. It is meaningful to explore the structure of GPRC5A and determine agonists or inhibitors to investigate its effectiveness in clinical trials for cancer treatment.

## Conclusions

Generally, the GPRC5A working model varies in different cancers. In this study, we demonstrated that crosstalk between the Hippo pathway and GPRC5A-cAMP-CREB signaling was critical for pancreatic cancer progression. This finding suggests that the GPRC5A-cAMP-CREB-YAP1 axis may provide candidate targets for therapeutic application.

## Supplementary Information

Supplementary Figure S1. Western blotting showed the expression of GPRC5A, YAP1 and CYR61 after transfecting YAP1 or control plasmids combined with LV-sh-NC or LV-sh-GPRC5A in PANC1 cells. One-way ANOVA, post hoc, Bonferroni, *p < 0.05, **p < 0.01, ***p < 0.001, (vs. LV-sh-NC+OE-NC); ##p < 0.01, ###p < 0.001, (vs. LV-sh-GPRC5A+ OE-NC); &p < 0.05, &&p < 0.01, (vs. LV-sh-NC+OE-YAP1). (TIF 4529 KB)

## Data Availability

All the datasets generated and analyzed in the present study are available from the corresponding author on reasonable request.

## Abbreviations

GPCRs: G-protein coupled receptors
GPRC5A: G-Protein coupled Receptor Class C, Group 5, Member A
PDAC: Pancreatic ductal adenocarcinoma
OS: Overall survival
DFS: Disease-free survival
cAMP: Cyclic adenosine monophosphate
DEGs: Differentially expressed genes
