# Supplementary figures and images for: Upregulated GPRC5A disrupting the Hippo pathway promotes the proliferation and migration of pancreatic cancer cells via the cAMP-CREB axis
Source: Discov Oncol. 2023 Feb 3;14:17. doi: 10.1007/s12672-023-00626-1 (PMC9898488; doi:10.1007/s12672-023-00626-1)

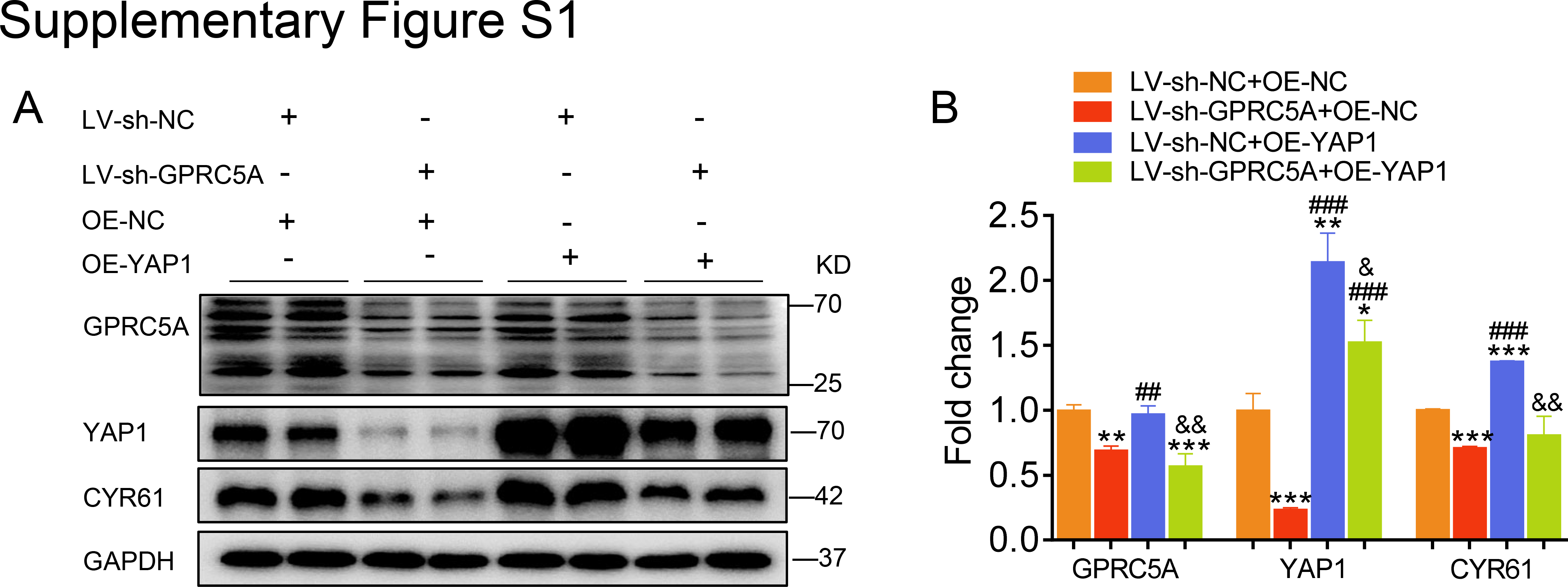

Supplement: Supplementary file 1 — Supplementary Figure S1. Western blotting showed the expression of GPRC5A, YAP1 and CYR61 after transfecting YAP1 or control plasmids combined with LV-sh-NC or LV-sh-GPRC5A in PANC1 cells. One-way ANOVA, post hoc, Bonferroni, *p < 0.05, **p < 0.01, ***p < 0.001, (vs. LV-sh-NC+OE-NC); ##p < 0.01, ###p < 0.001, (vs. LV-sh-GPRC5A+ OE-NC); &p < 0.05, &&p < 0.01, (vs. LV-sh-NC+OE-YAP1). (TIF 4529 KB) [file 12672_2023_626_MOESM1_ESM.tif]
